# IL-18 serum concentration is continuously elevated in typical familial Mediterranean fever with M694I mutation and can distinguish atypical type

**DOI:** 10.1186/1546-0096-13-S1-P80

**Published:** 2015-09-28

**Authors:** T Yamazaki, T Shigemura, N Kobayashi, K Honda, M Yazaki, J Masumoto, K Migita, M Tamura, K Agematsu

**Affiliations:** 1 Saitama Medical Center, Saitama Medical University, Pediatrics, Kawagoe, Japan; 2 Shinshu University Graduate School of Medicine, Infection and Host Defense, Matsumoto, Japan; 3 Shinshu University School of Medicine, Pediatrics, Matsumoto, Japan; 4 Northern Yokohama Hospital, Showa University, Children's Medical Center, Yokohama, Japan; 5 Shinshu University School of Medicine, Internal Medicine, Matsumoto, Japan; 6 Ehime University Graduate School of Medicine and Ehime Proteo-medicine Research Center, Pathology, Toon, Japan; 7 Nagasaki Medical Center, Clinical Research Center, Omura, Japan

## Objectives

Familial Mediterranean fever (FMF) can be classified into typical and incomplete/atypical types based on clinical findings and gene analysis, although biomarkers that distinguish typical from atypical FMF have not been unclear.

## Methods

We here investigated the serum cytokine profiles of IL-1β, IL-6, IL-8, TNF-α, IFN-γ, and IL-18 in FMF compared with those in Kawasaki disease.

## Results

IL-1β, IL-6, IL-8, TNF-α, and IFN-γ were not increased in either type of FMF in the remission state and in controls, and IL-6 was elevated during attack periods among patients. Serum IL-18 levels were significantly higher in typical FMF patients with M694I *MEFV* mutation in remission than in controls at the same level as flared Kawasaki disease, which further increased during attack periods. In contrast, IL-18 levels in atypical FMF with P369S-R408Q mutation or in typical FMF without M694I mutation was not increased, in either disease states.

## Conclusion

Thus, serum IL-18 levels at attack increase more than in remission, and that are an excellent marker to distinguish between the two types of FMF.

